# Acupuncture modulates temporal neural responses in wide brain networks: evidence from fMRI study

**DOI:** 10.1186/1744-8069-6-73

**Published:** 2010-11-02

**Authors:** Lijun Bai, Jie Tian, Chongguang Zhong, Ting Xue, Youbo you, Zhenyu Liu, Peng Chen, Qiyong Gong, Lin Ai, Wei Qin, Jianping Dai, Yijun Liu

**Affiliations:** 1 Medical Image Processing Group, Institute of Automation, Chinese Academy of Sciences, Beijing 100190, China; 2 School of Life Science and Technology, Xidian University, Xi'an 710071, China; 3 Beijing TCM Hospital affiliated to Capital University of Medical Sciences, Beijing 10010, China; 4 West China Hospital of Sichuan University, Sichuan 610041, China; 5 Department of Radiology, Beijing Tiantan Hospital, Capital University of Medical Sciences, Beijing, 100050, China; 6 McKnight Brain Institute, Departments of Psychiatry and Neuroscience, University of Florida, Gainesville, FL 32610, USA; 7 Department of Biomedical Engineering, Peking University, Beijing 100871, China

## Abstract

**Background:**

Accumulating neuroimaging studies in humans have shown that acupuncture can modulate a widely distributed brain network, large portions of which are overlapped with the pain-related areas. Recently, a striking feature of acupuncture-induced analgesia is found to be associated with its long-last effect, which has a delayed onset and gradually reaches a peak even after acupuncture needling being terminated. Identifying temporal neural responses in these areas that occur at particular time -- both acute and sustained effects during acupuncture processes -- may therefore shed lights on how such peripheral inputs are conducted and mediated through the CNS. In the present study, we adopted a non-repeated event-related (NRER) fMRI paradigm and control theory based approach namely change-point analysis in order to capture the detailed temporal profile of neural responses induced by acupuncture.

**Results:**

Our findings demonstrated that neural activities at the different stages of acupuncture presented distinct temporal patterns, in which consistently positive neural responses were found during the period of acupuncture needling while much more complex and dynamic activities found during a post-acupuncture period. These brain responses had a significant time-dependent effect which showed different onset time and duration of neural activities. The amygdala and perigenual anterior cingulate cortex (pACC), exhibited increased activities during the needling phase while decreased gradually to reach a peak below the baseline. The periaqueductal gray (PAG) and hypothalamus presented saliently intermittent activations across the whole fMRI session. Apart from the time-dependent responses, relatively persistent activities were also identified in the anterior insula and prefrontal cortices. The overall findings indicate that acupuncture may engage differential temporal neural responses as a function of time in a wide range of brain networks.

**Conclusions:**

Our study has provided evidence supporting a view that acupuncture intervention involves complex modulations of temporal neural response, and its effect can gradually resolve as a function of time. The functional specificity of acupuncture at ST36 may involve multiple levels of differential activities of a wide range of brain networks, which are gradually enhanced even after acupuncture needle being terminated.

## Background

Acupuncture has emerged as a common modality of alternative and complementary therapeutic intervention in the Western medicine. In spite of its public acceptance, an unequivocal scientific explanation regarding physiological and biological mechanisms underlying acupuncture has not been attained and awaits further investigations. One unresolved but fundamental question is whether acupuncture needling at certain acupoints can produce functionally specific effects in the brain compared to a sham or placebo control procedure.

Previous neuroimaging studies have revealed that acupuncture stimulation can elicit widespread cerebro-cerebellar brain regions [1-6], largely overlapping with the neural networks for both pain transmission and perception [7]. These regions process information in circuits that can broadly be assumed to engage: the affective (amygdala, hippocampus), sensory (thalamus, primary (SI) and secondary (SII) somatosensory cortices), cognitive (ACC, anterior insula), and inhibitory (PAG, hypothalamus) processing during the experience of pain [8]. Several studies on brain responses to acupuncture stimuli in patients with chronic pain or pain condition compared with controls have also found prominent signal attenuations in the amygdala and SI, as well as signal potentiations in the hypothalamus and motor-related areas [9-11]. Moreover, a very recent study has found that the underlying analgesia efficacy of acupuncture mainly involves the underlying molecular pathways, particularly by activating A1 receptor [12]. This evidence has brought to light the fact that the central representation of a peripheral acupuncture signal may involve a network of neurons, which are widespread distributions across multiple levels of brain areas.

To date, most of neuroimaging studies have primarily focused on the spatial distribution of neural responses to acute effects of acupuncture. However, the acupuncture needling itself is not sufficient to produce its analgesia effects [13]. Evidence from both human behavior and animal studies has indicated that a striking feature of acupuncture analgesia, in both human and animals, is its longevity--a delayed onset, gradual peaking and gradual returning [14-16]. For a typical 30-min acupuncture session, the pain threshold has a slowly increase tendency even outlasting the treatment [13]. We infer that acupuncture procedure typically involves two administration steps: (1) needling stimulation in deep tissue with skin piercing and biochemical reaction to tissue damage, and (2) prolonged effects after the removal of acupuncture needle stimulation [13,17-19]. It is also substantiated that the physical needling stimulus, as well as the delayed effect of acupuncture, can similarly activate many brain areas [18,19]. Careful interpretations of acupuncture intervention depend on how to effectively characterize the nature of temporal variations underlying neural activities that give rise to hemodynamic responses, rather than how to simply detect the occurrence of such changes.

Conventional statistical fMRI analysis of acupuncture has typically adopted the hypothesis-based approach (general linear model, GLM), and mainly tested whether activity in a brain region is systematically related to some known input function [3,5,6,20]. In other words, this model-based approach implicitly embodies specific assumptions or requires a priori knowledge about the shape of the time courses to be investigated. Since the temporal profile of acupuncture-associated response is difficult to specify in advance, the GLM approach is limited and may be susceptible to errors [17]. Only recently, independent components analysis (ICA), using few a priori assumption, is applied to extract reliable patterns underlying the psychological activity of acupuncture [19,21]. However, this method still lacks in accuracy to make direct inferences on whether a component (brain network) varies over time and when changes occur in certain time points. Great emphasis has, therefore, been given to understand temporal characteristics of these spatially defined brain regions, with considerations for how multiple levels of their dynamic activities in concert cause the processing of acupuncture.

Built upon our previous studies [17,18,22,23], we have formulated a hypothesis that distinct, time-dependent changes elicited by acupuncture are mirrored by the temporal responses observed within the wide brain networks, which has been suggested to participate in different stages of acupuncture process. To address this question, we adopted the control theory based approach namely change-point analysis, in which a hierarchical exponentially-weighted moving average (HEWMA) approach was used to make direct inferences on acupuncture-related activities [24,25]. This method can also effectively deal with high individual variabilities of neural responses induced by acupuncture [26].

## Results

### Psychological results

**Figure 1:**
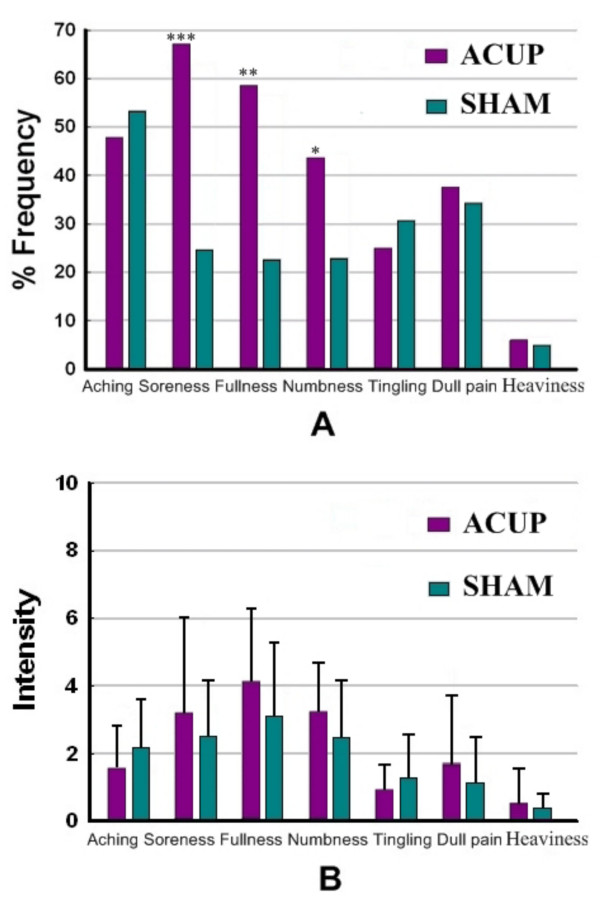
**Results of psychophysical deqi sensations**. A. The percentage of subjects who reported having experienced the given sensation (at least one subject experienced the seven sensations listed). Numbness, fullness, and soreness were found greater for acupuncture at ST36. B. The intensity of the reported sensations measured by an average score (with standard error bars) on a scale from 0 denoting no sensation to 10 denoting an unbearable sensation. The average stimulus intensities (mean ± SE) were approximately similar during acupuncture at ST36 (2.4 ± 1.7) and nonacupoint (2.2 ± 1.9).

The prevalence of subjective "deqi" sensations was expressed as the percentage of individuals in the group that reported the given sensations (Figure 1A). Numbness (acupuncture at ST36: 43.4% of subjects, acupuncture at nonacupoint: 22.1%, P < 0.01), fullness (acupuncture at ST36: 58.9%, acupuncture at nonacupoint: 21.7%, P < 0.005), and soreness (acupuncture at ST36: 68.3%, acupuncture at nonacupoint: 23.8%, P < 0.0005) were found greater for acupuncture at ST36 under Fisher's exact test. The intensity of sensations was expressed as the average score ± SE (Figure 1B). The levels of sensations were kept low (mild to moderate), and the averaged intensities (mean ± SE) were approximately similar for the acupuncture at ST36 (2.4 ± 1.7) and nonacupoint (2.2 ± 1.9) (P > 0.05). These results indicated that acupuncture at nonacupoint with same needling manipulation could effectively reduce the subjects' bias toward the stimulation.

### Condition-specific and temporal fMRI responses

During the needling manipulation period, group results from the GLM presented that acupuncture at ST36 evoked a remarkable predominance of signal increases in the wide limbic-subcortical regions (P < 0.005, uncorrected, cluster size >5 voxels, Table 1). This network included the insula, cingulate cortices, prefrontal cortex (PFC), SI/posterior parietal cortex (PPC), SII, thalamus, amygdala, hippocampus, cerebellum and brainstem structures. Following acupuncture at a non-acupoint (NAP), there were also marked positive responses but a relatively small extent of spatial distributions and less intensive signal changes. These regions were only localized in the thalamus, SI, SII, primary motor cortex (M1), PFC, anterior insula and cerebellum (Table 2). For both conditions, the estimated onset time from the change-point analysis ranged from around 25 TRs to around 91 TRs, indicating that these areas may activate differently induced by the peripheral acupuncture needling. Nonetheless, most brain regions responded around the time of the stimulation onset (around 43 TRs ~ 63 TRs). Notably, some regions, such as the posterior parietal cortex (PPC), middle cingulate cortex (MCC), insula and amygdala, became active even before the acupuncture manipulation. In point of fact, these areas may be critical for perceived intrusion or threat, vigilance-related judgment and decision on external stimuli processing [27].

**Table 1:** Four types of temporal neural responses with differential onsets and durations following acupuncture at ST36 (coordinate and *t *score of peak voxel, P < 0.005, uncorrected; TR = 2s).

Time-varied response		ACUP					
		
		Talairach					
		
Transient Response	Hem/BA	x	y	z	t value	Onset TRs	Duration TRs
Middle Cingulate Cortex	R/32	3	19	35	7.62	29	40
Posterior Cingulate Cortex	R/29	6	-46	16	4.58	51	35
Thalamus							
VMpo	R	15	-20	7	4.71	31	37
MDvc	R	6	-20	4	11.31	27	48
VPL	L	-15	-17	6	9.31	33	29
Posterior Parietal Cortex	R/7	22	-49	63	5.88	32	36
SI	R/2	65	-22	23	9.03	34	41

Bimodal Response							

Perigenual ACC	R/32	3	44	9	5.15	43	216
Amygdala	L	-21	-4	-17	4.30	38	250
Hippocampus	R	27	-15	-12	5.15	54	243
Posterior Insula	L	-56	-34	18	8.90	38	243
Putamen/Claustrum	L	-21	-5	-9	10.45	49	175
Inferior Parietal Cortex	R/40	62	-34	24	12.23	91	239
Cerebellum	L	-12	-80	-21	11.52	63	232

Intermittent Activity							
Red Nucleus/SN	L	-6	-18	-2	8.76	47	122
Periaqueductal Gray	R	3	-30	-12	5.83	75	162
RVM	R	6	-26	-38	5.23	77	156
Hypothalamus	R	3	-3	-7	5.36	57	148
SMA	R/6	4	-16	54	4.68	45	131

Sustained Activity							

Anterior Insula	R/13	39	20	2	8.50	34	332
SII	L/40	-65	-25	21	8.63	37	241
Prefrontal Cortex	L/47	-36	17	-6	10.01	41	254

Abbreviations: BA-brodmann area; Hem-hemisphere; VMpo-ventromedial posterior nuclear complex; MDvc-ventrocaudal mediodorsal nucleus; VPL-ventroposterior lateral; SI-primary somatosensory cortex; ACC-anterior cingulate cortex; SN-substantia nigra; RVM-rostral ventromedial medulla; SMA-supplementary motor cortex; SII-secondary somatosensory cortex; PFC-prefrontal cortex; L-Left; R-Right.

**Table 2:** There types of temporal neural responses with differential onsets and durations following acupuncture at nonacupoint (coordinate and *t *score of peak voxel, P < 0.005, uncorrected; TR = 2s).

Time-varied Response		SHAM					
		
		Talairach					
			
Transient Response	Hem/BA	x	y	z	t value	Onset TRs	Duration TRs
Putamen	L	-24	-3	-5	3.15	34	42
Thalamus-VPL	R	20	-20	5	3.64	37	78
SII	R/40	56	-14	17	3.59	25	62
Posterior Parietal Cortex	R/7	18	-46	63	3.13	27	27

Bimodal Response							

SI	L/2	-56	-24	40	4.15	21	370
Premotor Cortex	L/4	-59	-10	33	3.43	68	202
Inferior Parietal Cortex	R/40	65	-30	32	3.95	57	133
Cerebellum -Pyramis	L	-12	-69	-32	4.62	44	112

Sustained Activity							

Anterior Insula	R/13	34	22	5	3.76	37	270
Prefrontal Cortex	L/9	-30	34	35	4.51	43	245
Orbitofrontal Cortex	L/11	-6	65	-3	4.27	33	261

Abbreviations: BA-brodmann area; Hem-hemisphere; VPL-ventral posterior lateral nucleus; SI-primary somatosensory cortex; SII-secondary somatosensory cortex; L-Left; R-Right.

**Figure 2:**
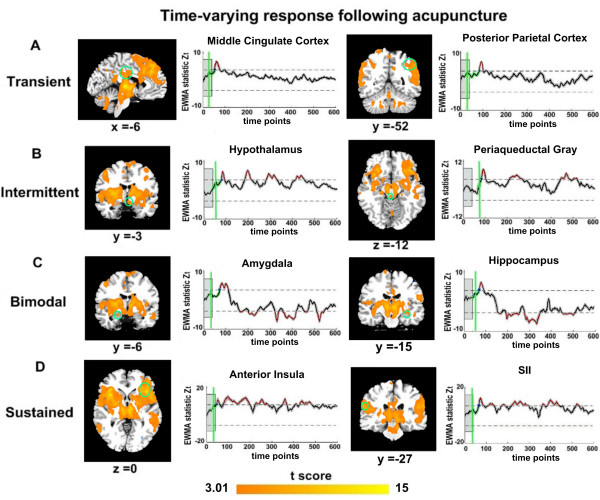
**Differential temporal neural responses induced by acupuncture at ST36 as a function of time**. The baseline period was indicated by the shaded gray box, and the EWMA-statistic was shown by the thick black line (corrected over time and FDR corrected at *α *= 0.05 over space), with gray shading denoting the standard error across participants. The estimated CP for onset activity was presented in green line. The control limits were shown by dashed lines. Abbreviations: SII-secondary somatosensory cortex.

Examination of the time courses of these activated regions (ROIs) revealed distinctive temporal signatures. The activation durations varied from transient activities (around 29 TRs or 51 TRs) to sustained activities (around 241 TRs or 332 TRs), suggesting a complex temporal dynamics underlying acupuncture at ST36 rather than a simple variation under the experimental-controlled amplitude. Specifically, four types of temporal neural responses emerged as a function of time. The transient activity was mainly located in the MCC, PPC and thalamus, which apparent activity reached its maximum soon after the onset of stimulation and terminated immediately following the offset of stimulation (Figure 2A). In contrast, the PAG and hypothalamus also exhibited transient activity, but presented more intermittent patterns across the length of the scanning (Figure 2B). Besides, sustained activity particularly occurred in the anterior insula and PFC (Figure 2D), suggesting their roles in exerting continuous control and coordinated influence during the whole process. Although other regions also displayed relatively durative activities, the signal responses showed more prominently time-dependent patterns--signal increased during the stimulation period (around 40 TRs ~ 100 TRs), and decreased steadily to reach the statistical significance exceeding an opposite control limit. These regions were mainly located in the amygdala, hippocampus, pACC, posterior insula, putamen and cerebellum (Table 1 and Figure 2C). This bidirectional response may reflect a significant modulation of activity amplitudes during different stages of the acupuncture (the needling administration and post- stimulus period).

**Figure 3:**
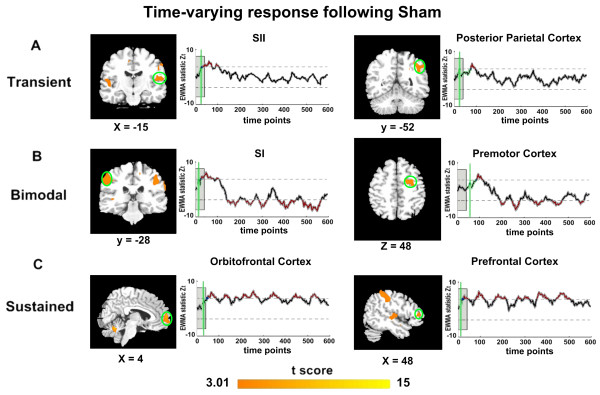
**Differential temporal neural responses induced by acupuncture at nonacupoint as a function of time**. The baseline period was indicated by the shaded gray box, and the EWMA-statistic was shown by the thick black line (corrected over time and FDR corrected at *α *= 0.05 over space), with gray shading denoting the standard error across participants. The estimated CP for onset activity was presented in green line. The control limits were shown by dashed lines. Abbreviations: SII-secondary somatosensory cortex. Abbreviations: SI-primary somatosensory cortex; SII-secondary somatosensory cortex.

Compared with acupuncture at ST36, there were generally three types of temporal neural responses following acupuncture at nonacupoint, which have a very limited range of spatial distributions (shown in Figure 3). There were transient activities in the PPC and thalamus, decreased tendency (bimodal) in the M1 and cerebellar areas, and sustained response in the orbitofrontal cortex (OFC) and dorsolateral PFC (DLPFC). Apart from these condition-specific neural involvements, even in the same areas, the temporal profile also exhibited totally distinct patterns between different conditions. Of particularly interest were remarkably different modulations between the SI and SII. The SII presented sustained and positive signal changes following the acupuncture at ST36, but transient activities limited to the needling manipulation period in the acupuncture at nonacupoint group; in contrast to the SI showing the transient facilitation during the acupuncture at ST36, a salient inhibition primarily occurred following acupuncture at nonacupoint.

## Discussion

In the current study, we found that acupuncture could induce the dynamic responses in the wide brain areas in which there was a variety of onset time and different durations of induced neural activities. Identifying such changes that occur at a particular time period as well as its temporal profile may shed lights on how such peripheral acupuncture inputs are conducted and mediated through a neurophysiological system of the pain processing. Results showed that neural responses evoked by acupuncture needling presented consistently positive signal changes, but more complex temporal responses during the post-acupuncture action period. Such time-dependent neural responses derived from the change-point analysis indicated different engagements of neural mechanisms, such as a decreased tendency in the amygdala and hippocampus, intermittent increased activities in the hypothalamus and PAG, sustained responses in the insula and PFC. In addition, neural responses involving acupuncture at ST36 and nonacupoint were heterogeneous: the more time prolonged, the more differences were found in the corresponding neural activities. The current investigation of time-dependent brain responses to the genuine acupuncture may provide new information regarding its neurobiological basis.

### Differential patterns during different stages of acupuncture

Previous neuroimaging studies, using the block-designed paradigm, provide little knowledge to evaluate neural responses during different stages of acupuncture action -- isolating the concurrent brain activity related to the sensory stimulation from the brain activity associated with the prolonged effect resulting from the same stimulation. For the above-mentioned weakness, the current research adopted a NRER design paradigm in order to dissociate neural responses under different stages of acupuncture. Our findings presented that simply acupuncture needling can evoke consistently increased signal changes in the wide brain networks, but more complex and time-dependent neural responses during the post-stimulus phase. One possible explanation is that acupuncture needling, like kind of painful stimulus, generally involves a needling stimulation in deep tissue with both skin piercing and biochemical reactions to the tissue damage; this predominant experience may be mainly associated with excitatory responses in pain-related areas. As the effect of acupuncture may require a period of time to develop, its complex action on disassemble neural system may occur as time prolonged.

### Temporal neural responses following acupuncture at ST36

Compared with acupuncture at nonacupoint, acupuncture at ST36 can induce more complex response patterns with a larger extent of spatial distributions and relatively more robust magnitudes (shown in Table 1 and Table 2), such as the intermittent activity in the brainstem structures (PAG, RVM) and hypothalamus. The activations of these nuclei were consistent with the findings from animal experiments, which supported the notion that acupuncture afferent pathways engaged the structures of the descending antinociceptive system [28-30]. In addition, we speculate that acupuncture may inhibit the neural activity in the pain-intensity encoding regions as time prolonged, including the posterior insula, putamen/claustrum and cerebellum. This finding was in a great extent consistent with the main conclusion from Kong et al that verum acupuncture can significantly inhibit the brain response to calibrated pain stimuli, as indicated by fMRI signal decreasing in the same structures [31]. Therefore, the inhibition of these areas may be related to the effect of acupuncture on the modulation of chronic pain.

Apart from both facilitation and suppression of these brain activities, verum acupuncture can also induce complex bidirectional response patterns in the amygdala, hippocampus and pACC - excitatory responses to acupuncture needling but decreased to the below baseline during the post-stimulus period (shown in Table 1). Particularly, the above-threshold signal changes in the amygdala showed an early start (onset = 38 TR) even before the onset of acupuncture manipulation, reflecting the emotional response (anxiety) associated with an impending stimulus event. As the needling manipulation terminated, the neural response inverted into an opposite direction with a long-lasting duration. This regulation plasticity of the amygdala, consistent with the reciprocal relationship between pain and negative affect, not only contributes to the generation and enhancement of pain responses, but also modulates pain processing through the descending inhibitory control system [32]. Our results, with primarily negative BOLD response in these limbic-related areas, are also supported by accumulating neuroimaging acupuncture studies [2,6,19], and one study further indicates that such signal attenuation in the amygdala is correlated with the elevation of pain threshold in subjects [10]. Considering that reduction in negative emotions may be important to analgesia effect [33], the amygdala, with its well-documented role in affective states and related disorders, appears well positioned to play an important role in acupuncture analgesia by the emotion modulation.

Another interesting finding was that the anterior insula presented sustained neural activations through the whole scanning. Previous studies have also supported that the anterior insula is the most consistently observed findings and reported regardless of acupoint location or acupuncture mode [3,5,6,17,18,26]. Converging evidence from many literatures implicates insula as the most reliable region in brain imaging studies on pain [34], and considers it as a limbic integration cortex for complex and preprocessed sensory information with direct association with the SI, SII, prefrontal areas and amygdala, which are important sources of hippocampus and ACC afferents [35-38]. These available results support the proposition that the anterior insula may be involved in acupuncture action as a key modulator to control the ongoing interactions among key nociceptive processing brain regions.

### Heterogeneous sensory responses to acupuncture at ST36 and nonacupoint

Previous investigations, focusing on the spatial distribution of neural responses to acute effects of acupuncture within a relatively short-term span, have argued that possible neural differences between two conditions are too subtle for detection in fMRI [1,5,26]. As the effects of acupuncture may require a period of time to develop, we speculated that the differences may only emerge over time when its delayed effect was being studied. As observed in our findings, acupuncture at ST36 and nonacupoint shared a similar activation pattern in the somatosensory areas (SI and SII) during the needling manipulation period. However, more dynamic and disentangled neural responses emerged during the post-acupuncture resting period -- sustained activation of the SII following verum acupuncture in contrast to salient inhibition of the SI following SHAM. From this observation, we inferred that the role of somatosensory areas may be heterogeneous to these two stimulus interventions. Accumulating evidence has illustrated that inhibited neural activity in the SI may be due to the intense or repetitive mechanical stimulations of a peripheral nerve [39-41]. On the other hand, the SII, aside from encoding the sensory-discriminative aspects of pain like the SI, is more involved in higher levels of pain cognitive-evaluative components, such as recognition, learning, and memory of painful events [42,43]. Some evidence also indicates that the averaged fMRI activation level of the SII, rather than the SI, is positively correlated with acupuncture-induced analgesic effect across the subjects [10]. Therefore, the SII, with its long-lasting neural response, may disclose its pivot role in characterizing the central expression of acupuncture effects, serving not only sensory aspects but more high-level modulation functions. Different neural responses in the somatosensory areas may enlighten us a new way insightful enough into different sensory effects induced by the same acupuncture stimulation at different anatomical site, possibly due to the distribution of distinct peripheral sensory receptors and nerve fibers.

With our designations, the difference between acupuncture at ST36 and nonacupoint was limited to the needling points. Therefore, the comparison of these two conditions was expected to reveal the acupoint-specific response in the human brain [44]. Although there were remarkable overlapping brain regions involving acupuncture on both ST36 and nearby non-acupoint, the brain networks were more intrinsically heterogeneous and consisted of neural subsystems as time prolonged. We inferred that a greater proportion of the impulses generated by SHAM may reach the somatosensory cortex and frontal cortices to exert its activating effect, which may support the clinical facts that acupuncture at sham points can also provide partial analgesia in chronic pain [45]. In contrast, brain networks underlying acupuncture at ST36 seemed to be more extensive, and we speculated that these multiple neural circuitries were themselves under dynamic controls by suppressing the action in both pain-affective areas and incoming noxious information, as well as mobilizing the antinociceptive action in the inhibitory system.

## Conclusions

In conclusion, the current fMRI study using control theory based approach namely change-point analysis has led to the possibility of understanding complex mechanisms by which the peripheral acupuncture stimuli and neural dynamics were interrelated as a function of time. Our results have provided evidence to support that brain networks underlying different acupuncture interventions (needling at a real acupoint vs. at a non-acupoint) were heterogeneous, especially at a prolonged stage following acupuncture. We postulated that acupuncture needling at ST36 may trigger the peripheral nociceptive information processing and that acupuncture effect may engage multiple pain-ascending pathways distributed in the limbic and brainstem substrates. On the other hand, acupuncture at a nonacupoint primarily activates the somatosensory and frontal association cortices. In sum, acupuncture at ST36 may have specific temporal modulations on neural responses of the wide brain networks involved in pain.

## Methods

### Experimental paradigm

In order to reduce intersubject variabilities, all the subjects were recruited from a homogeneous group of 16 college students (8 male, ages of 22.5 ± 1.8, mean ± SD). Subjects were all acupuncture naïve, and right-handed according to the Edinburgh Handedness Inventory [46]. Subjects were screened and excluded for major medical illnesses, head trauma, neuropsychiatric disorders, intake of prescription medications within the last month, and any contraindications for exposure to a high magnetic field. After a complete description of the study was given to all subjects, written informed consent was obtained; the research protocol was approved by the West China Hospital Subcommittee on Human Studies. The experiment was also conducted in accordance with the Declaration of Helsinki.

Two fMRI runs were conducted in this study: (1) acupuncture before, during and after acupuncture administration at an acupoint ST36; and (2) SHAM for a nearby nonacupoint (2-3 cm apart from ST36). Subjects were not informed of the order in which these two runs would be performed, and were asked to keep their eyes closed to prevent from actually observing the procedures. During the scanning, subjects were also told to remain relaxed without engaging in any mental tasks. According to subjects' reports after the scanning, they affirmed to keep awake during the whole scanning. The presentation sequence of these two protocols was randomized across the fMRI runs, and the order of presentation was counterbalanced across subjects. Every subject was exposed to only one fMRI run per day in order to mitigate any potential long-lasting effects following the acupuncture administration [18].

**Figure 4:**
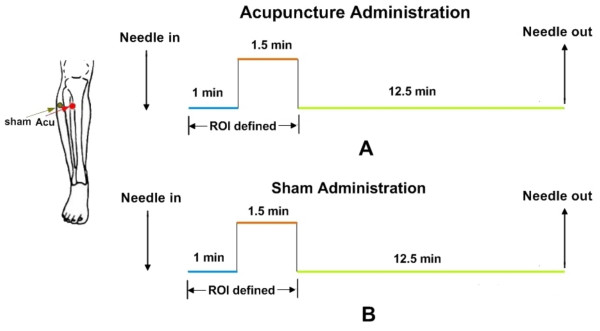
**Experimental design paradigm**. Acupuncture needle manipulation was performed at an acupoint ST36 (Zusanli, arrow pointing to red dot) or a nonmeridian-point focus approximately 2-3 cm distant laterally (NMP, arrow pointing to green dot) on the right leg, respectively. Functional run incorporated the NRER paradigm, incorporating 1.5 min needle manipulation, preceded by 1 min rest epoch and followed by 12.5 min rest scanning.

Acupuncture was performed at an acupoint ST 36 on the right leg (Zusanli, located four finger breadths below the lower margin of the patella and one finger breadth laterally from the anterior crest of the tibia). This is one of the most frequently used acupoints for pain analgesia and disorders of multiple systems [45]. Acupuncture stimulation was delivered using a sterile disposable 38 gauge stainless steel acupuncture needle, 0.2 mm in diameter and 40 mm in length. The needle was inserted vertically to a depth of 2-3 cm, and administration was delivered by a balanced "tonifying and reducing" technique [17]. Stimulation consisted of rotating the needle clockwise and counterclockwise for 1 min at a rate of 60 times per min. So far most fMRI studies on the brain correlates of acupuncture have adopted block designs with repeated stimuli, which are obviously less suited to study the temporal features of brain responses. Moreover, in block designs it is difficult to disentangle the concurrent brain activity related to the needling manipulation from the brain activity associated with its delayed effect resulting from the same stimulation. Here, a new experimental paradigm, namely the NRER fMRI design, was employed. The NRER-fMRI scanning lasted 15 min per run, including 1.5-min needling manipulations, preceded by a 1-min rest and followed by another 12.5 min rest scanning (see Figure 4). The procedure was performed by the same experienced and licensed acupuncturist on all subjects.

Recently a number of noninvasive sham controls have been developed and tested [3,47,48]. While these hold promise in some respects, they also have limitations in what they can be used for, thus they should be used only when it is clear that their use matches the question for which sham treatment model is being selected [17,49]. Sham acupuncture is proved to a reasonable placebo control in many acupuncture fMRI setting, and can effectively reduce the subjects' bias toward the stimulation [6,50]. In the current study, we also employed sham acupuncture as a control model. Sham acupuncture was initially devised by an experienced acupuncturist, which performed at a nonmeridian point (2-3 cm apart from ST36) with needle depth, stimulation intensity, and manipulation method identical to those used in the ST36.

At the end of each fMRI scanning, the subjects completed a questionnaire that used a 10-point visual analogue scale (VAS) to rate their experience (or "deqi") of aching, pressure, soreness, heaviness, fullness, warmth, coolness, numbness, tingling, dull or sharp pain they felt during the scan. The VAS was scaled at 0 = no sensation, 1-3 = mild, 4-6 = moderate, 7-8 = strong, 9 = severe and 10 = unbearable sensation [3]. The questionnaire also had one blank row for subjects to add their own words if the above descriptors did not embody the sensations they experienced during the stimulation. Because sharp pain was considered an inadvertent noxious stimulation, we excluded the subjects from further analysis if they experienced the sharp pain (greater than the mean by more than two standard deviations). Among the sixteen participants, none experienced the sharp pain.

### Data acquisition and analysis

The images were acquired on a 3T GE Signa scanner. A custom-built head holder was used to prevent head movements. Thirty-two axial slices (FOV = 240 mm × 240 mm, matrix = 64 × 64, thickness = 5 mm), parallel to the AC-PC plane and covering the whole brain were obtained using a T2*-weighted single-shot, gradient-recalled echo planar imaging (EPI) sequence (TR = 1500 ms, TE = 30 ms, flip angle = 90°). Prior to the functional run, high-resolution structural information on each subject was also acquired using 3D MRI sequences with a voxel size of 1 mm^3 ^for anatomical localization (TR = 2.7 s, TE = 3.39 ms, matrix = 256 × 256, FOV = 256 mm × 256 mm, flip angle = 7°, slice thickness = 1 mm).

All preprocessing steps were carried out using statistical parametric mapping (SPM5, http://www.fil.ion.ucl.ac.uk/spm/). The images were first slice-timed and then realigned to correct for head motions (none of the subjects had head movements exceeding 1 mm on any axis and head rotation greater than one degree). The image data was further processed with spatial normalization based on the MNI space and re-sampled at 2 mm × 2 mm × 2 mm. Finally, the functional images were spatially smoothed with a 6 mm full-width-at-half maximum (FWHM) Gaussian kernel. The statistics were color-coded and mapped in Talairach space [51].

### Hierarchical exponentially-weighted moving average (HEWMA) analysis

Previous neuroimaging studies have generally adopted the voxel-wise GLM approach to detect the neural responses induced by acupuncture. This model is well-suited for testing whether variability in a voxel's time course can be explained by a set of a priori defined regressors that model predicted responses to psychological events of interest; whereas it becomes invalid when the psychological states have uncertain onset times, temporal intensity profiles, and durations. Inasmuch as the actual temporal characteristic of acupuncture cannot be specified a priori, such model-based analysis may be susceptible to estimate errors [17]. The present work aimed to investigate the temporal profiles of the neural activities underlying acupuncture and at the same time addressing the methodological limitations described above by adopting the HEWMA approach. This approach requires little specification of a priori constraints on the dynamic inference and lends itself to decipher different pain-related areas to their specific role in the acupuncture intervention. Moreover, this data-driven approach is proved to be more appropriate for group fMRI data, particularly when it fails to replicate experimental manipulations within subjects.

**Figure 5:**
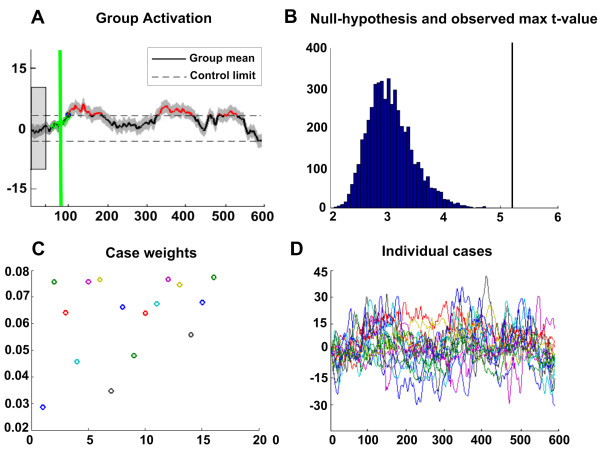
**The detailed scheme for HEWMA analysis**. A. Group results of the rostral ventromedial medulla generated from the HEWMA analysis. The estimated change-point (CP) for onset activation was indicated as green line. Grey shading presented the standard error of EWMA statistic. B. The corrected p-value generated from the Monte Carlo simulations. Black line: observed max T; distribution: null hypothesis max T. C. Case weights equaled to the inverse of total variation (including within-subject and between-subject variation) for certain subject. Weights are based on variability during the baseline interval, meaning that higher variance may result in a lower weight for that subject. D. The individual time courses for the 16 subjects.

The EWMA analysis models the *f*MRI time course as a mixture of two normal distributions, as *X*_0 _~ *N*(*θ*_0_, σ^2^) for the baseline state (in-control), and *X*_1 _~ *N*(*θ*_1_, σ^2^) for the activated state (out-of-control, OOC). Through testing the change in the data (fMRI time course) distribution (as the null hypothesis *θ*_0 _= *θ*_1_), one can determine whether the state of a psychological event varies deviations from baseline. The EWMA statistic *Z*_*t *_is defined as a weighted average of the current and all past observations, *z*_*t *_= *λx*_*t *_+ (1 - *λ*)*z*_*t*-1 _(*t *= 1,...*n*). The inferences about this statistic deviations from baseline follows as $T_{t}=|Z_{t}-\theta_{0}|/\sqrt{Var(Z_{t})}$, and the corresponding control limits (confidence intervals) can be calculated as $\theta_{0}\pm t*\sqrt{Var(Z_{t})}$. If the EWMA statistic *Z*_*t *_exceeds the control limits, the state change occurs (i.e., the state of activation has changed). The next step is to estimate when exactly such a change took place, i.e., estimate the unknown parameter *τ *(the change point, CP). Presumably there are commonly several state changes in the *f*MRI time series, one may adopt a Gaussian mixture model to determine each observation as either belonging to the baseline or the activated (OOC) distribution for detecting the multiple CPs. Through this procedure, the length of time spent in the activated state can be estimated, as the number of OOC points (detailed information presented in reference [24]). Obviously, the procedure outlined above can only be used at an individual level. In order to perform a mixed-effects analysis on *f*MRI group data, the weighted (hierarchical) regression is defined as the weights inversely proportional to the total variance for each subject (hierarchical EWMA, HEWMA). In the framework, one can also get corrected P-values by performing the Monte-Carlo simulation [24]. The detailed scheme of HEWMA analysis is shown in Figure 5.

### Temporal profile of acupuncture-related activities estimated by change-point algorithm

In the current study, a multi-step analytic approach was used to identify brain regions associated with different temporal characteristics elicited by the acupuncture. The general linear model (GLM) was firstly used to identify specific regions of interest (ROIs) related with the acupuncture stimulation. Given that the post-stimulus period may still contain acupuncture-associated effects, the mean signal intensity of the rest epoch preceded by the active stimulation served as the baseline [17]. Thus, the geographical extents of the ROIs were based on the t-contrast of the baseline and the acupuncture stimulation condition (P < 0.005, uncorrected, spatial-extent of 5 contiguous voxels). For bilaterally activated regions, the hemisphere anatomical area with a more significant t value was selected for further analysis. Confounding effects of fluctuations were removed across the averaged time course within each ROI: *(i)*. to minimize the effect of global drift, voxel intensities were scaled by dividing the value of each time point by the mean value of the whole-brain image at that time point; *(ii)*. several sources of spurious or regionally nonspecific variance were then removed by regression including: six parameters obtained by rigid body head motion correction, the signal averaged over the whole-brain, the lateral ventricles, and the deep cerebral white matter. Consequentially, a three-step process was undertaken: *(i)*. the time course within each ROI of individual subject was firstly analyzed with the EWMA analysis [24]. In this study, we used the AR (2) model to calculate the EWMA statistic and its variance to mitigate the periodic noise oscillations (e.g., pulsatile motion due to breathing and cardiac activity) in fMRI data. *(ii)*. considering inter-subject variation on startups (or delays) of the BOLD signal responses, population inferences were estimated through a weighted regression, as the weights inversely proportional to the total variance for each subject http://www.columbia.edu/cu/psychology/tor/. *(iii)*. by using a zero-crossing method and Gaussian mixture model, the next step in the analysis entails estimating when exactly the change takes place, as well as the activation duration (transient or sustained activity). The initial CP (onset) was estimated by the zero-crossing method, and activity durations were calculated using the Gaussian mixture model described above (*α *0.05, FDR corrected).

## List of abbreviations

fMRI: functional magnetic resonance imaging; SI: primary somatosensory cortex; SII: secondary somatosensory cortex; ACC: anterior cingulated cortex; PAG: periaqueductal gray; GLM: general linear model; ICA: independent components analysis; HEWMA: hierarchical exponentially-weighted moving average; NMP: non-acupoint; M1: primary motor cortex; PFC: prefrontal cortex; PPC: posterior parietal cortex; MCC: middle cingulate cortex; ROIs: regions of interest; pACC: perigenual ACC; OFC: orbitofrontal cortex; DLPFC: dorsolateral prefrontal cortex; RVM: rostral ventromedial medulla; NRER: non-repeated event-related; VAS: visual analogue scale; EPI: echo planar imaging; FWHM: full-width-at-half maximum; OOC: out-of-control; CP: change point;

## Competing interests

The authors declare that they have no competing interests.

## Authors' contributions

LJB carried out the experiment and wrote the manuscript. JT made substantial contributions to the design and coordination of this study. CGZ provided fMRI methodology in the study. TX participated in the design of this study. YBY performed the statistical analysis of this study. ZYL participated in the data processing. PC performed the entire acupuncture procedure. QYG have made substantial contributions to the acquisition of data. LA participated in the design and coordination of this study. WQ participated in the analysis and interpretation of data. JPD have made substantial contributions to the design and acquisition of data. YJL made substantial contributions to the conception and design, have been involved in drafting the manuscript and revised it critically for important intellectual content. All authors read and approved the final manuscript.
